# Temperature Evolution of Composition, Thermal, Electrical and Magnetic Properties of Ti_3_C_2_T_x_-MXene

**DOI:** 10.3390/ma17102199

**Published:** 2024-05-08

**Authors:** Shreyas Srivatsa, Waldemar Tokarz, Janusz Przewoźnik, Tomasz Strączek, Krzysztof Grabowski, Paweł Rutkowski, Tadeusz Uhl, Jan Kulawik, Dariusz Kata, Dominika Madej, Jerzy Lis, Czesław Kapusta

**Affiliations:** 1 Space Technology Centre, AGH University of Krakow, 30-059 Krakow, Poland; sshreyas@agh.edu.pl (S.S.); kgrabow@agh.edu.pl (K.G.); tuhl@agh.edu.pl (T.U.); 2 Faculty of Physics and Applied Computer Science, AGH University of Krakow, 30-059 Krakow, Poland; januszp@agh.edu.pl (J.P.); kapusta@agh.edu.pl (C.K.); 3 National Synchrotron Radiation Centre SOLARIS, Jagiellonian University, Czerwone Maki 98, 30-392 Kraków, Poland; tomasz1.straczek@uj.edu.pl; 4 Department of Robotics and Mechatronics, AGH University of Krakow, 30-059 Krakow, Poland; 5 Faculty of Materials Science and Ceramics, AGH University of Krakow, al. A. Mickiewicza 30, 30-059 Krakow, Poland; pawel.rutkowski@agh.edu.pl (P.R.); kata@agh.edu.pl (D.K.); dmadej@agh.edu.pl (D.M.); lis@agh.edu.pl (J.L.); 6 Kraków Division, Łukasiewicz Research Network—Institute of Microelectronics and Photonics, 30-701 Kraków, Poland; jan.kulawik@ite.waw.pl

**Keywords:** MXenes, titanium carbide, low temperature, thermal, magnetoresistance, space applications

## Abstract

MXenes are a family of two-dimensional nanomaterials. Titanium carbide MXene (Ti_3_C_2_T_x_-MXene), reported in 2011, is the first inorganic compound reported among the MXene family. In the present work, we report on the study of the composition and various physical properties of Ti_3_C_2_T_x_-MXene nanomaterial, as well as their temperature evolution, to consider MXenes for space applications. X-ray diffraction, thermal analysis and mass spectroscopy measurements confirmed the structure and terminating groups of the MXene surface, revealing a predominant single OH layer character. The temperature dependence of the specific heat shows a Debye-like character in the measured range of 2 K–300 K with a linear part below 10 K, characteristic of conduction electrons of metallic materials. The electron density of states (DOS) calculations for Ti_3_C_2_OH-MXene reveal a significant DOS value at the Fermi level, with a large slope, confirming its metallic character, which is consistent with the experimental findings. The temperature dependence of electrical resistivity of the MXene samples was tested for a wide temperature range (3 K–350 K) and shows a decrease on lowering temperature with an upturn at low temperatures, where negative magnetoresistance is observed. The magnetoresistance versus field is approximately linear and increases its magnitude with decreasing temperature. The magnetization curves are straight lines with temperature-independent positive slopes, indicating Pauli paramagnetism due to conduction electrons.

## 1. Introduction

In recent years, with the advent of new nanomaterials, technological developments using material science studies have attained greater focus. Carbon-based nanomaterials [1] dominated such technological developments initially, but with the reporting of new nanomaterials like boron nitride, molybdenum disulfide and MXenes, the scope for technology development has enlarged. The unique physical properties of these nanomaterials are being utilized for sensing various quasi-static and dynamic phenomena of field variables like temperature, strain, pressure and magnetic flux. Such sensing devices based on nanomaterials are being designed for the next phase of human aspiration—outer space. The challenges of human exploration of outer space involve not just exposure to harsh environments compared to Earth, but also a need for new technologies to function in such environments. Sensor development also needs to catch up with this growing aspiration [2]. And the foundation for any such technological development would be understanding how the nanomaterials work for a wide operating range of field variables (first in ground-based studies) such that they can measure and be sustained in outer space. With this motivation, the present paper focuses on studying the temperature evolution of the composition and low-temperature electrical, thermal and magnetic properties of an MXene nanomaterial, namely titanium carbide MXene.

MXenes are a family of two-dimensional (2D) nanomaterials discovered in 2011 [3]. The first compound synthesized by etching aluminum from the titanium aluminum carbide MAX (Ti_3_AlC_2_-MAX) phase compound was titanium carbide MXene (Ti_3_C_2_T_x_-MXene). The general convention used for MXenes in the literature is M_n+1_X_n_T_x_, where M refers to transition metals (like Ti, V, Mo); X refers to carbides, nitrides or carbonitrides; and T_x_ refers to the functional groups (like O, F, OH, Cl) that terminate the surface of MXenes (here n = 1–4). The physical properties and chemical stability of Ti_3_C_2_T_x_-MXene have been explored since its synthesis, and a few of the impressive properties are film-forming ability, good elastic strength [4,5], high conductivity [6], fast response to dynamic loads [7], electromagnetic shielding [8], biocompatibility [9], low toxicity [10] and low infrared emissivity [11]. These properties have led to many applications like sensor development for structural health monitoring (SHM) [12], wearable health monitoring devices [13], neurobiological sensing [14], multifunctional composite materials [15], antennas [16], energy harvesters [17] and storage devices [18]. The study of various physical properties of Ti_3_C_2_T_x_-MXene is still under progress in the research community. With development of many active and passive electronics with MXenes (also referred to as MXetronics in the literature [19]) like dielectric capacitors [20], supercapacitors [21,22,23], transistors [19], memristors [24], sensors [12], triboelectric nanogenerators [25] and piezoelectric electronics [26], the performance of MXenes in various operating environments is critical for their applications in space technology. As new applications of MXenes are being envisaged for the space domain, a thorough experimental study of their thermal and magnetic properties becomes imminent. Such a study provides a base for tailoring the functionalities of MXene nanomaterials for various use cases in space exploration.

In this study, Ti_3_C_2_T_x_-MXene nanomaterial was synthesized using the widely followed etching method with the combination of lithium fluoride and hydrogen chloride. This was followed by a minimally intensive layer delamination process that allowed delaminated Ti_3_C_2_T_x_-MXene monolayers to be obtained [27,28]. The complete synthesis and processing method is described in Section 2.1. The colloidal solution of delaminated Ti_3_C_2_T_x_-MXene was then vacuum-filtered and vacuum-dried to form MXene films or powders that were used for investigations in this study. Following this, various methods were employed to study the material characteristics and various physical properties of Ti_3_C_2_T_x_-MXene.

The structure of the paper is as follows: Initially, the Ti_3_C_2_T_x_-MXene material synthesis and processing steps are described along with various experimental methods employed in this paper. Then, the results of the X-ray diffraction method employed to confirm the successful obtaining of Ti_3_C_2_T_x_-MXene are presented. This is followed by the first experimental reporting of the temperature dependence (2 K to 300 K) of specific heat of Ti_3_C_2_T_x_-MXene. These experimental studies are complemented by theoretical calculations of the electronic density of states to validate some of the crucial physical properties. Then, the thermal analysis data along with quadrupole mass spectroscopy results are presented and discussed. Also, the thermal analysis of aged MXene material, which is new in the literature, is shown. Then, the temperature dependence of electrical resistivity of the MXene material is analyzed. A wide range of magnetic fields (−90 kOe to 90 kOe) was applied to study the magnetoresistance behavior and magnetization curves of the material. Finally, the impact of these physical properties of Ti_3_C_2_T_x_-MXene on space technology applications is discussed. The experimental studies of the physical properties of Ti_3_C_2_T_x_-MXene in this paper are a precursor for the exploration of new technology development for outer space exploration and for SHM of space structures.

## 2. Materials and Methods

### 2.1. Ti_3_C_2_T_x_-MXene Synthesis and Processing

**Figure 1:**
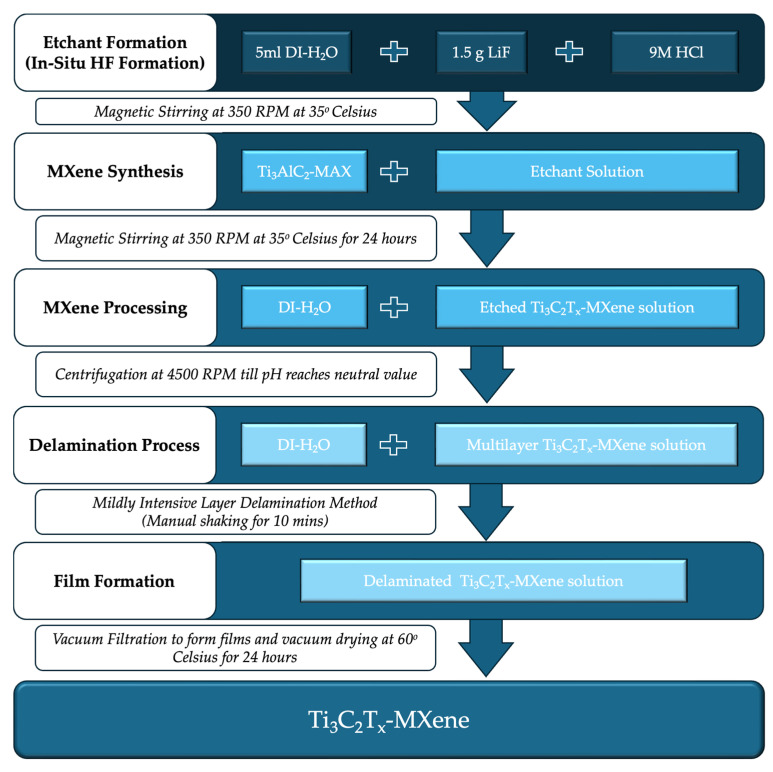
Process diagram of the material synthesis steps of Ti_3_C_2_T_x_-MXene used in this paper.

Ti_3_C_2_T_x_-MXene was synthesized by in situ hydrogen fluoride formation with a mixture of lithium fluoride (LiF) and hydrogen chloride (HCl) and using the minimally intensive layer delamination (MILD) method. This is similar to the guidelines provided in the literature works [7,27]. First, 1 g of titanium aluminum carbide (Ti_3_AlC_2_-MAX compound; ≤40-micron particle size; supplier: Materials Research Center, Kiev, Ukraine) was added slowly over 5 min into the etchant solution (with the addition of 9 M HCl, Honeywell Fluka, Charlotte, NC, USA; 1.5 g of LiF, Sigma-Aldrich, Burlington, MA, USA; and 5 mL of deionized water, Chempur, Seattle, WA, USA) to etch aluminum from Ti_3_AlC_2_-MAX phase compound. The etching reaction was allowed to continue for 24 h with a stirring speed of 350 rpm at 35 °C [29,30]. Once the reaction was complete, the suspension was washed with deionized water by centrifugation at 4500 rpm to cause the powder to settle and decant the clear supernatant. The washing cycles were repeated until the supernatant reached a neutral pH value. This MXene suspension contained multilayer Ti_3_C_2_T_x_-MXene. The MILD method was employed, involving vigorous shaking for 10 min to obtain a delaminated Ti_3_C_2_T_x_-MXene suspension. The suspension then vacuum-filtered to form Ti_3_C_2_T_x_-MXene films or powder with a vacuum drying process at 60 °C for 24 h before being used for the experiments presented in this paper. The process diagram of the material synthesis steps is presented in Figure 1.

### 2.2. X-ray Diffraction Study

X-ray diffraction (XRD) was applied to determine the crystal structure of the material. It was performed in Bragg–Brentano geometry on a Panalytical Empyrean X-ray diffractometer with Cu K_α_ radiation on the Ti_3_AlC_2_-MAX and Ti_3_C_2_T_x_-MXene to confirm the etching of aluminum from the MAX phase compound to form a 2D MXene.

### 2.3. Specific Heat Measurements

The heat capacity measurements were carried out by a two-tau relaxation method with the heat capacity option of a Quantum Design PPMS-9 during heating in the temperature range of 1.85 K–280 K. The Ti_3_C_2_T_x_-MXene samples were attached and thermally coupled to addenda with Apiezon N grease. A background signal from the addenda and grease was recorded versus temperature as a control result after each other measurement was carried out.

### 2.4. Thermal Analysis Experiments

The thermal analysis was performed with the use of differential scanning calo-rimetry DSC-TG using two simultaneous thermal analyzer (STA) models: STA 449 F3 Jupier (Netzsch company, Selb, Germany) at the Faculty Laboratory of Thermophysical Research at Faculty of Material Science and Ceramics AGH University of Krakow and STA 449 F3 Jupiter equipped with quadrupole mass spectrometer QMS 403 C Aelos (Netzsch). The measurement was carried out in an alumina crucible with a lid. The heating rate was 10 K/min from RT to 1073 K in argon flow. Three types of data were recorded: thermal effect heat flow, thermogravimetry and released gases from the sample. The OH, H_2_O, H_2_, F and Cl released from the sample were analyzed as a result of MAX phase chemical etching and liquid composition for 2D material preparation. The results were interpreted in terms of MXene terminating groups and their influence on the physical properties of MXenes.

### 2.5. Electrical Resistivity and Magnetoresistance Measurements

The temperature dependence of electrical resistivity was studied with a Quantum Design Physical Property Measurement system (QD PPMS), San Diego, CA, USA, with the Thermal Transport and Resistivity option. The system is equipped with a 90 kOe superconducting magnet, and it can reach temperatures from 2 K up to 400 K for these options. The sample was powdered and pressed into a Teflon tube with two copper rods providing continuous compressive force and therefore good thermal and electrical conductivity. The copper rods were pressed into the Teflon tube at a kilobar pressure until the resistance between them dropped to the range of hundred ohms. Also, the magnetoresistance, i.e., the dependence of the electrical resistivity on the applied magnetic field up to 90 kOe, was studied at selected temperatures.

### 2.6. Magnetometric Measurements

The magnetometric experiments were carried out on the QD PPMS with the Vibrating Sample Magnetometer (VSM) option in the temperature range from 3 K to 300 K. The magnetic hysteresis loop measurements were carried out in the magnetic field range from −90 to 90 kOe.

## 3. Results

### 3.1. X-ray Diffraction Study

**Figure 2:**
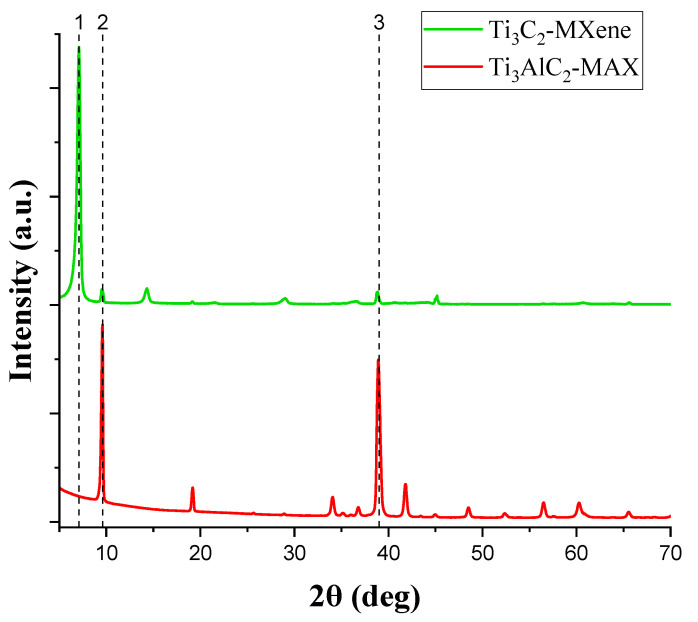
XRD results of Ti_3_AlC_2_-MAX phase and Ti_3_C_2_T_x_-MXene. Line 1 represents the shift of peak (002) of Ti_3_C_2_-MXene after the etching of Ti_3_AlC_2_-MAX shown by line 2. Line 3 indicates the removal of Al from the Ti_3_AlC_2_-MAX after etching.

The experimental details of the X-ray diffraction (XRD) measurements are provided in Section 2.2. The confirmation of the successful etching of the Ti_3_AlC_2_-MAX to form Ti_3_C_2_T_x_-MXene is discussed based on the XRD analysis and with reference to literature studies [3]. Figure 2 shows the XRD results for both Ti_3_AlC_2_-MAX and Ti_3_C_2_T_x_-MXene. Line (dotted) 3 indicates the 39° peak of the Al-containing plane of the Ti_3_AlC_2_-MAX which vanishes upon etching as described in Section 2.1. Along with the removal of Al, the (002) peak of Ti_3_AlC_2_-MAX shifts from 9.50° to 7.10° for Ti_3_C_2_T_x_-MXene, indicated by line 2 and line 1 (both dotted), respectively. The shifting of the peak also indicates the intercalation of water (OH groups) within delaminated Ti_3_C_2_-MXene, and for the present case of 7.10°, the presence of one hydroxyl layer (monohydrated with d-spacing ~12.4 Å) within the delaminated Ti_3_C_2_T_x_-MXene is considered [31]. The (002) peak might vary its position between 5° and 7° depending on the relative humidity of the MXene samples after being subjected to the vacuum-filtration and vacuum-annealing process [31]. This confirms the successful synthesis of Ti_3_C_2_T_x_-MXene [27,28]. The XRD analysis of these Ti_3_C_2_T_x_-MXene films resulted in only (00l) peaks (where l = 2, 4, 6, etc.) (as per the convention followed by the MXene research community), as shown in Figure 2.

### 3.2. Specific Heat Measurements

**Figure 3:**
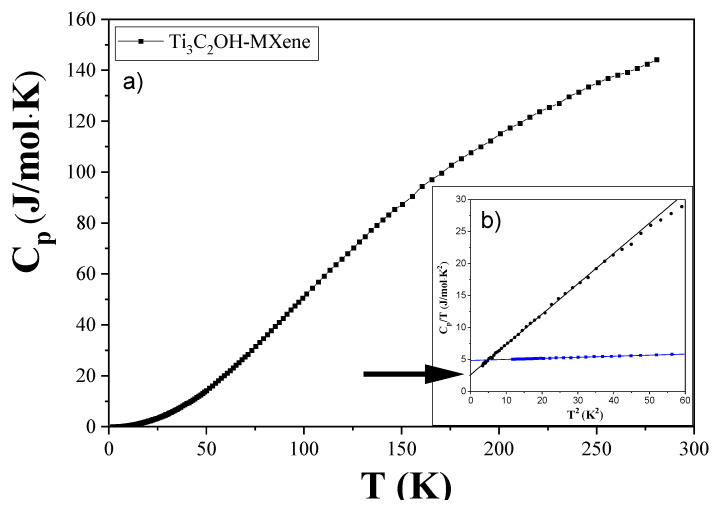
(**a**) Temperature dependence of specific heat for Ti_3_C_2_T_x_-MXene. (**b**) The inset shows the low T range as C_p_/T versus T^2^ plots for Ti_3_C_2_T_x_-MXene (black line) and Ti_3_AlC_2_-MAX (blue line). Linear fits to the data in the inset (solid lines) were used to estimate the Debye temperature and Sommerfeld coefficient (shown with black arrow) of the compounds.

The details of the specific heat measurement experiment are provided in Section 2.3. The temperature dependence of the specific heat (Cp) for the Ti_3_C_2_T_x_-MXene compound is shown in Figure 3a,b. The smooth curve indicates that no phase transition occurs through the temperature range of measurement. To determine the Debye temperature (Θ_D_) and Sommerfeld (γ) coefficient conveniently, we approximated the Cp/T versus T^2^ plot shown in Figure 3b. A comparison between the Ti_3_C_2_T_x_-MXene and Ti_3_AlC_2_-MAX compounds is made by also placing the results of specific heat measurements of the Ti_3_AlC_2_-MAX compound in this figure. The electronic specific heat (Sommerfeld) coefficient of Ti_3_C_2_T_x_-MXene is non-zero (coordinate of black line crossing the vertical axis in Figure 3b). This confirms its metallic nature, as predicted theoretically in [32], but the surface termination will affect its properties, and this in turn is dependent on the synthesis and processing methods employed [33]. The values of Θ_D_ and γ parameters, namely Θ_D_ = 160.1(3) K and γ = 2.62(4) J/(mol·K^2^) obtained in our study, are compared to those determined for Ti_3_AlC_2_-MAX compound, Θ_D_ = 484.2(1.6) K and γ = 4.848(6) J/(mol·K^2^) [34], respectively. The much lower Debye temperature of Ti_3_C_2_T_x_-MXene indicates a much softer lattice structure of this 2D nanomaterial than that of the parent MAX phase compound. The quantitative value of specific heat obtained in our study for Ti_3_C_2_T_x_-MXene (~142 J mol^−1^K^−1^) at 273 K is more than an order of magnitude higher than that of graphene (~7 J mol^−1^K^−1^) [35,36].

### 3.3. Density of States Calculations

**Figure 4:**
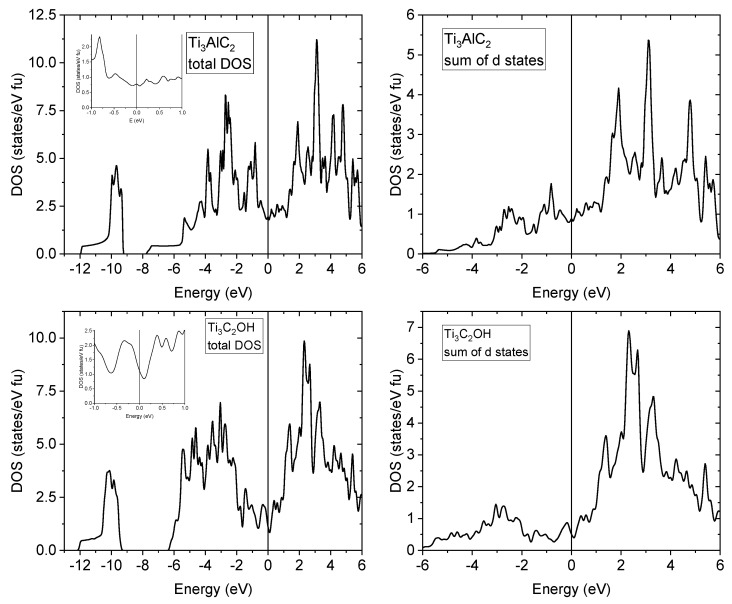
The total density of states (DOS; left side) and the sum of partial d states (right side) are shown with the Ti_3_AlC_2_-MAX phase compound in the top row and Ti_3_C_2_OH in the bottom row.

**Figure 5:**
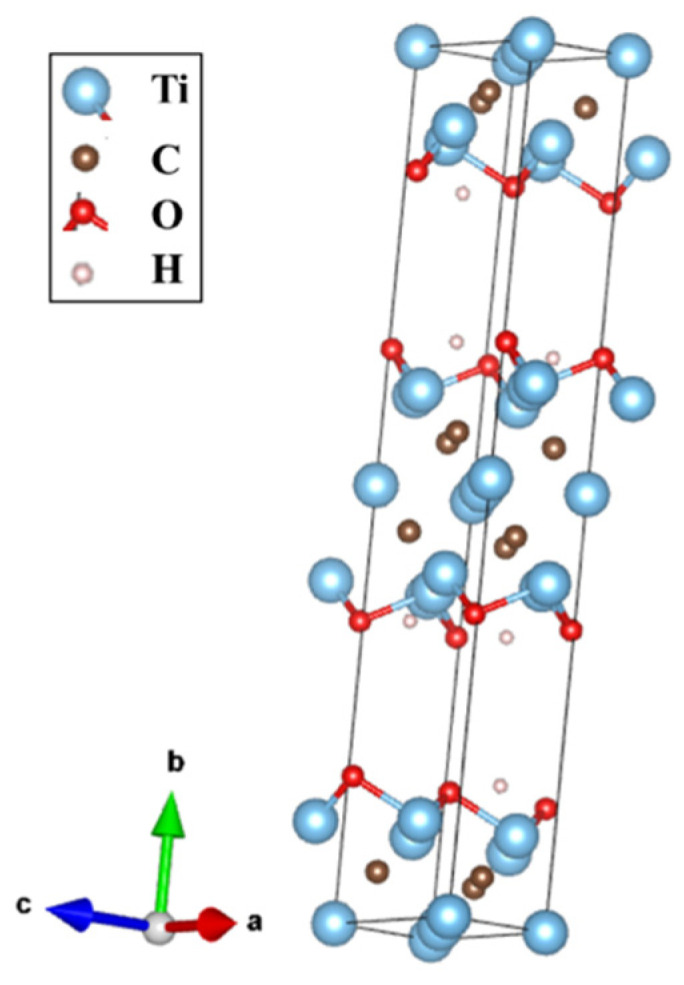
Optimized structure of Ti_3_C_2_ with [40] prepared for DFT-GGA calculations to obtain the electronic density of states.

The density of states (DOS) calculations were performed in full-potential WIEN2K code [37] based on density functional theory (DFT) [38,39] and the generalized gradient approximation (GGA) [40]. Based on the results presented in the next paragraphs, the Ti_3_C_2_OH stoichiometry was assumed. This is supported by the mass spectroscopy study showing an order of magnitude higher content of OH groups than other terminating groups and a single-layered termination resulting from the XRD study. The graphs in Figure 4 present the total and the projected ‘d’ DOS of Ti_3_AlC_2_-MAX and Ti_3_C_2_OH (structure shown in Figure 5 [41]) utilizing P63/mmc (ITC 194) and Pnnm (ITC 58) accordingly. The sum of ‘d’ states for Ti_3_C_2_OH was obtained by substitution of the OH group in place of Al in the MAX phase, and both structures were adjusted by minimizing energy as a function of volume and lattice parameters. Some Wyckoff positions with free parameters are calculated as minima of forces on their nuclei. As a result, we can notice a significant difference in the slope of the total DOS at the Fermi level and similar main ‘d’ states’ contribution to the total DOS. These calculations also are confirmed by the theoretical literature work on DOS in [32]. Comparing the resistivity and magnetoresistance of the Ti_3_AlC_2_-MAX phase and the Ti_3_C_2_OH-MXene, we realize that despite the similar DOS values, due to the two-dimensionality of the MXene, the mean free path of the conduction electrons can be much shorter, corresponding to a much higher resistivity. This would also correspond to a smaller positive magnetoresistance related to the “curling” of the conduction electrons in the applied magnetic field than in the respective MAX phase. As the “slopy” shape of DOS at the Fermi level in the Ti_3_C_2_OH-MXene can result in a considerable spin polarization, which can cause a negative contribution to the magnetoresistance; this tentatively explains its overall negative sign observed for our Ti_3_C_2_T_x_-MXene.

### 3.4. Thermal Analysis

**Figure 6:**
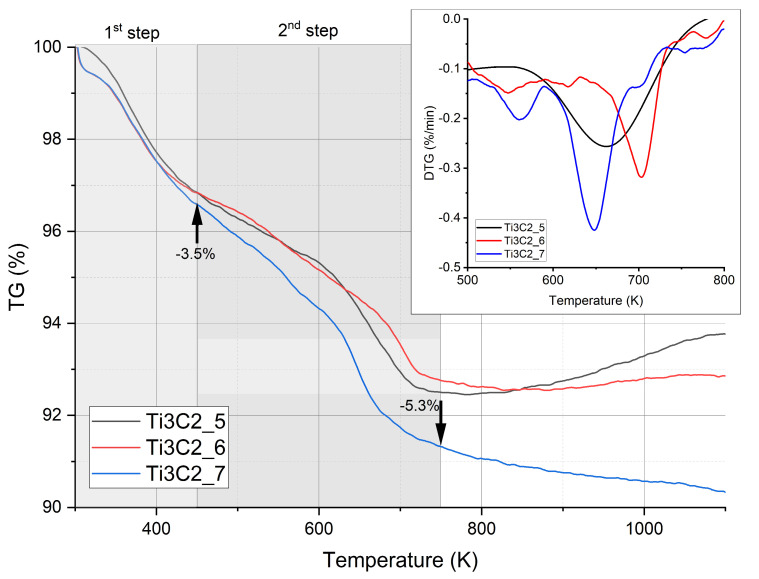
TG-DTG analysis of various MXene samples.

After the filtration process, the samples of MXenes were subjected to DSC-TG analysis in argon flow to check the mass change of the samples as a function of temperature. The results are presented in Figure 6. The thermogravimetric curves of three different Ti_3_C_2_T_x_-MXene samples confirm two steps of material mass loss that correspond to water desorption at low temperatures up to 450 K and the release of OH (hydroxyl) groups at higher temperatures, 450–750 K. The mass loss proceeds as follows: the first step is 3.5%, and the second step is up to 5.3%. At temperatures between 550 and 750 K, it is clearly visible that there is mostly OH group release and probably hydrogen as well, which is indicated by the first derivative of the TG curve (Figure 6). It was also confirmed in [42]. It was found in [42] that the first step is 2.7% mass loss, which relates to physically adsorbed water release. The second step, 1.4%, and third step, 2.3%, were explained by the release of CO_2_, OH groups and F. In the third step, for two samples in our experiments, it is observed that there is an increase in the sample mass even by 1.2%—Figure 4. This sample behavior at high temperatures can be explained by [43,44]. Liu [43], who measured the material in air conditions, describes this effect as a reaction of titanium with oxygen above 750 K and the generation of CO_2_. This process accelerates above 870 K, which correlates with our data [43]. In our case, oxygen can come from terminating OH groups. Li [44] divided the whole TG curve of analysis in argon flow into three stages: first concerning 0.38% mass loss up to 473 K, second 4.48% for the 473–1073 K range and third 2.47% for 1073–1273 K. He showed only a decrease in sample mass (TG curve) and stated that from 473 K, quasi-2D MXene structures are formed, which was confirmed by XRD/SEM analysis [44]. Above 1273 K, this 2D material can react with oxygen impurities coming from argon or OH groups to form titanium oxide [44]. Li [44] also mentioned that if there is oxygen and F termination, the TiOF_2_ compound can be formed in the range of 473–1273 K. This compound could originate from Ti_3_C_2_F_2_OH formed in the reaction with absorbed H_2_O and F coming from HF etching agent [44], which degrades above 473 K first to Ti_3_C_2_F_2_O_0.5_ due to water removal and in the second step to Ti_3_C_2_F_2_ with the release of oxygen and fluorine.

**Figure 7:**
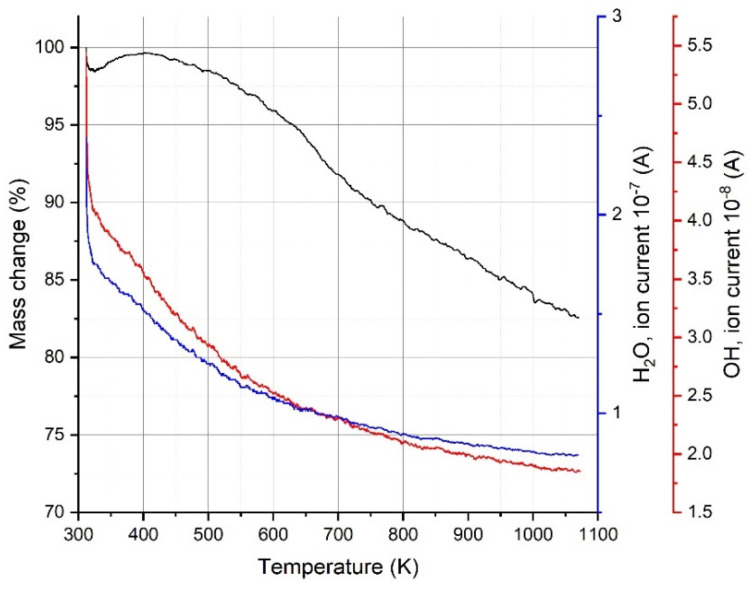
QMS results for H_2_O and OH groups released from the sample.

**Figure 8:**
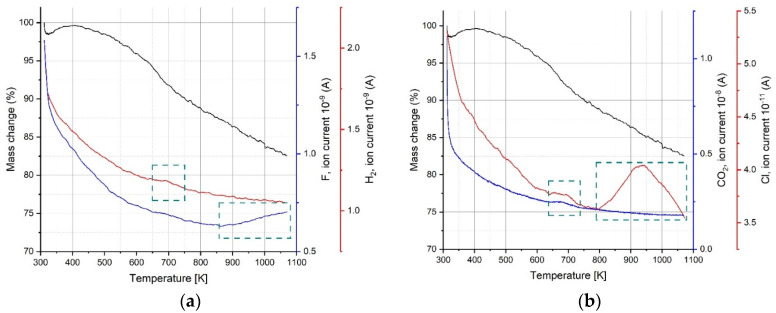
QMS results for gases released from the sample: (**a**) F and H_2_; (**b**) Cl and CO_2_. Pecular regions are marked and discussed in the text.

In order to determine what is happening during the heating of the MXene, the gases coming from the sample were analyzed by quadrupole mass spectroscopy (QMS). The QMS data of as-received samples are presented in Figure 7 and Figure 8. The removed water effect is visible as inflection in Figure 7, and OH groups are gradually removed from the sample with increasing temperature (higher current signal). The data presented in Figure 8 indicate a release of hydrogen and CO_2_ at around 700 K. This correlates with Feng’s experiments [42] and, also importantly, with a small amount of CO_2_ evolved at 650 K recorded by QMS (Figure 8b) (above 300 K in Liu paper). The QMS analysis also shows a signal of Cl and F evolved gases at temperatures above 800 K, and these gases are related to remnants of the lithium fluoride and hydrogen chloride etching agents (Figure 8a,b). The fluorine detected in our measurements was also reported by other researchers [42,43,44], but no QMS results were presented. The literature work [44] indicates that the release of oxygen is easier than that of fluorine due to bond energy, which suggests that in our case, it can have an influence on CO_2_ (Figure 8b) and TiO_2_ formation (Figure 7). In our case, we record F release (QMS data, Figure 8a) at about 220 K lower temperature than in the study of Li [44]. A comparison of the overall release intensities for the individual species shows the predominance of H_2_O and OH by two orders of magnitude in our MXene material, so the assumption of the Ti_3_C_2_OH structure for the DFT-GGA calculations seems to be justified, and this can be further used for theoretical calculations of the density of states. The mass loss at temperatures above 450 K, which is mostly connected with H_2_O and OH group release, explains the change in electrical resistivity of MXenes heated to the temperature 350 K in the cyclic measurement program with heating up and cooling down steps presented in figure in Section 3.5.

**Figure 9:**
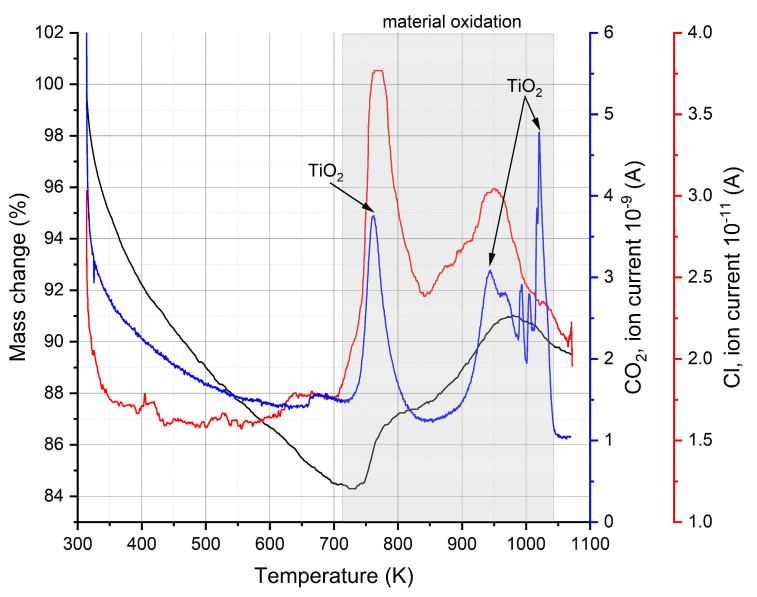
QMS results for gases released from the sample after 6 months.

The thermogravimetric analysis illustrated in Figure 6 in the case of one sample showed a 1.3% mass gain above 800 K. In order to check what occurs in MXene material upon aging, the sample was thermally analyzed after 6 months of aging under room conditions, and the DSC/TG-QMS results are shown in Figure 9. There is visible evolved Cl gas but also large quantities of CO_2_, which is an effect of MXene oxidation. Oxidation was also recorded on the thermogravimetric curve above 700 K. A pronounced two-step oxidation can be assigned to the aging process and the corresponding water adsorption. There are mainly two strong peaks observed in both the CO_2_ and Cl current signals, but the first CO_2_ peak is slightly shifted in temperature towards that of Cl. This means that the first mass gain and second mass gain correspond to TiO_2_ formation from MXene with CO_2_ gas release—as reported by Liu [43]. During oxidation, Li [44] observed the formation of anatase, then rutile, and CO_2_. He proposed a reaction scheme where Ti_3_C_2_F_2_OH with O_2_ gives products TiO_2_ and CO_2_. Those literature data confirm our mass spectroscopy analysis results presented in Figure 9. The only difference is that Li observed a decrease in the sample mass, and we have a mass gain above 700 K. The adsorption of water and the effect of oxygen on the MXene samples could result in the detection of relative humidity levels as well as water content in an environment where measurement is performed. This makes a case for the possibility of humidity sensors and water presence detectors with MXene sensors in the future.

### 3.5. Electrical Resistivity Measurements

**Figure 10:**
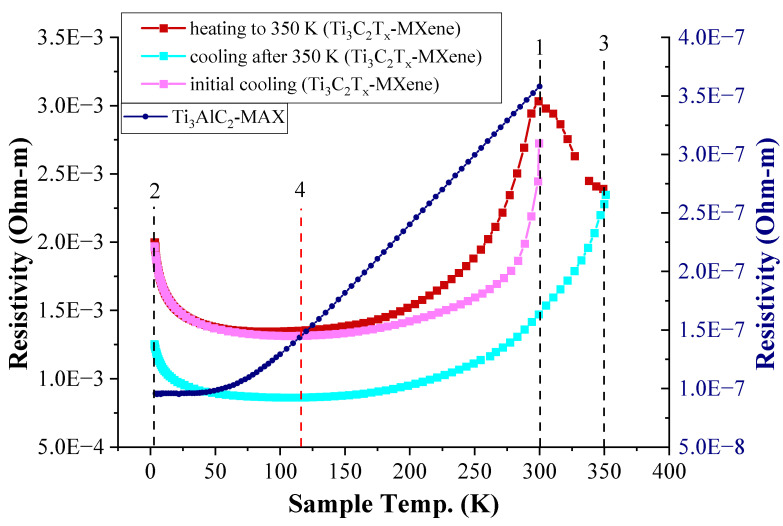
Temperature dependence of the resistivity of the Ti_3_C_2_T_x_-MXene sample (left Y-axis) (cycle of cooling–heating–cooling) and Ti_3_AlC_2_-MAX phase sample (right Y-axis).

The experimental setup for the measurement of the temperature dependence of the electrical resistivity of Ti_3_C_2_T_x_-MXene is described in Section 2.5. Figure 10 reports the variation in the electrical resistivity of the Ti_3_C_2_T_x_-MXene sample for a continuous temperature change between 3 K and 350 K. This is compared with the measurement of the variation in the electrical resistivity of the Ti_3_AlC_2_-MAX phase sample. The temperature dependence of the electrical resistivity of the Ti_3_AlC_2_-MAX phase is linear above 75 K with a positive slope indicating a metallic character of the material. For the Ti_3_C_2_T_x_-MXene sample, the slope of the temperature dependency of the electrical resistivity curve is negative below 115 K and positive above it. The downturn of the curve at about 300 K on subsequent heating indicates the effect of temperature on the desorption of hydroxyl groups or water molecules intercalating or terminating with delaminated Ti_3_C_2_T_x_-MXene during the vacuum-filtration process. These experimental observations also confirm and validate the temperature-dependent electrical resistivity behavior of the Ti_3_C_2_T_x_-MXene sample reported in the literature work [45].

The sample of Ti_3_C_2_T_x_-MXene was cooled down to 3 K from 300 K (points 1 to 2 in Figure 10). Then, the sample was heated to 350 K from 3 K (points 2 to 1 to 3 in Figure 10) and again cooled down to 3 K (points 3 to 2 in Figure 10). This single-cycle annealing process indicates a change in the slope of the curve of temperature dependency of the electrical resistivity of Ti_3_C_2_T_x_-MXene for the initial cooling and heating process. The heating of the Ti_3_C_2_T_x_-MXene sample up to 350 K in vacuum conditions in the experimental setup results in a reduction in electrical resistivity due to the removal of hydroxyl groups. This result confirms a similar observation made with the annealing of a Ti_3_C_2_T_x_-MXene sample in the literature in [46]. Further, the temperature cycling would follow the same pattern of a reduction in resistivity with each annealing cycle, thereby providing possible inputs for learning algorithms as part of new-age sensors that can be connected to real-time data-driven learning systems for field variable measurement [47,48,49].

The results presented in the section are for stacked delaminated Ti_3_C_2_T_x_-MXene with a vacuum-filtration process, and the positive slope of the resistivity curve is typical for a metal-type response above 115 K [50]. The low-temperature behavior with a negative slope of resistivity indicates semiconductor-like behavior (below 115 K). The magnetoresistance measurements discussed in Section 3.6 shed more light on the low-temperature behavior of Ti_3_C_2_T_x_-MXene (indicating negative magnetoresistance due to weak localization). It is also interesting to note that the changes in the slope of the electrical resistivity curve, depending on temperature cycling, could be employed to develop devices with switching behavior. Sensors for cryogenic chambers and low-temperature space environments can only be constructed from this material for a limited temperature range of operation (3 K–100 K) due to the variation in the slope that occurs close to 115 K (indicated by the dotted line at point 4 in Figure 10).

### 3.6. Magnetoresistivity and Magnetization Measurements

**Figure 11:**
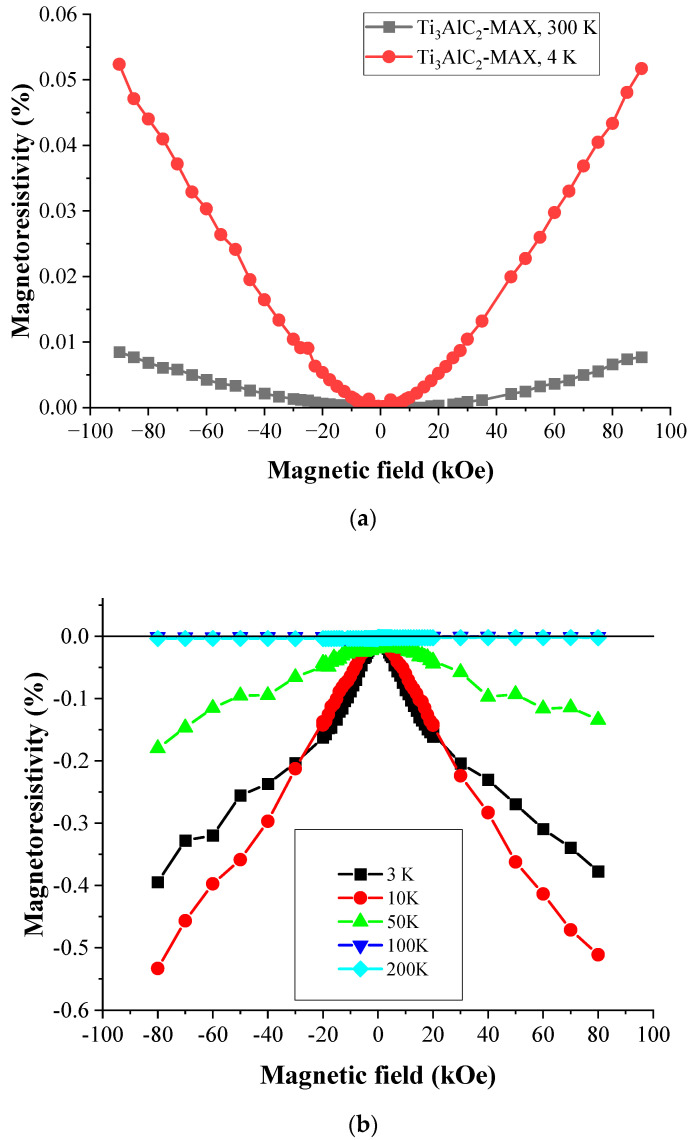
(**a**) The magnetic field dependence of the magnetoresistivity of the Ti_3_AlC_2_-MAX sample. (**b**) The magnetic field dependence of the magnetoresistivity of the Ti_3_C_2_T_x_-MXene sample.

The details on the experimental setup for the magnetoresistance measurement are provided in Section 2.6. The Ti_3_C_2_T_x_-MXene sample shows a negative magnetoresistance behavior below 100 K. This is in contrast with Ti_3_AlC_2_-MAX phase material [51], as shown in Figure 11a,b. An increase in the magnitude of MXene magnetoresistance with decreasing temperature is observed, indicating its possible origin linked to spin-dependent effects. The present results are provided for various temperatures below 300 K, while only a single 10 K magnetoresistance measurement has been reported in the literature [45]. It also indicates a negative magnetoresistance of Ti_3_C_2_T_x_-MXene.

**Figure 12:**
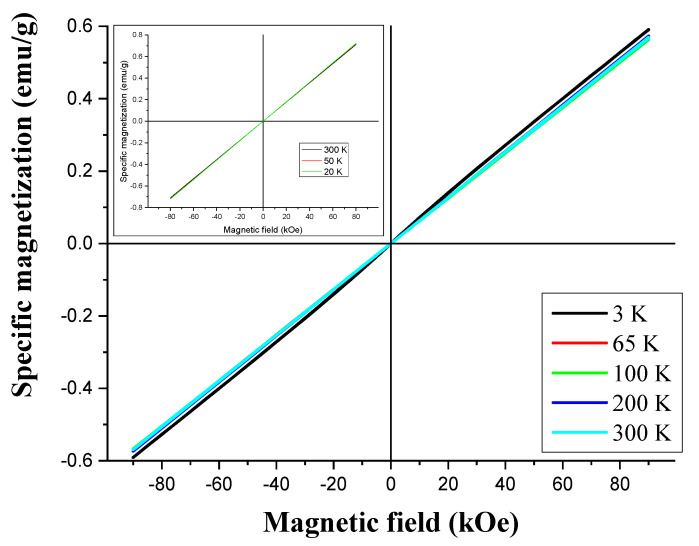
Magnetization curves of Ti_3_C_2_T_x_-MXene compound for various temperatures. Inset with magnetization curves of Ti_3_AlC_2_-MAX phase compound. Both with subtracted holder diamagnetism.

The magnetization curves obtained for Ti_3_C_2_T_x_-MXene are shown in Figure 12. They are straight lines with positive, temperature-independent slopes and no coercivity. This reveals temperature-independent magnetic susceptibility, characteristic of Pauli paramagnetism, attributed to conduction band electrons (similarly to the Ti_3_AlC_2_ Max phase that also shows Pauli paramagnetism; see the inset of Figure 12). This indicates the metallicity of our Ti_3_C_2_T_x_-MXene, confirming the specific heat results and the observation made in the literature as well [52]. The available theoretical study shows that pure MXenes could be ferromagnetic [31], but this literature paper neglected the surface termination of Ti_3_C_2_T_x_-MXene naturally present after chemical synthesis.

The presence of impurities due to magnetic stirrer usage during the etching process might lead to ferromagnetic behavior of Ti_3_C_2_T_x_-MXene, and for this reason, care should be taken during the synthesis and processing stage of Ti_3_C_2_T_x_-MXene. The effect of surface termination on this electrical property needs more explorations for better control of sensor devices envisaged using MXenes, although initial work has already been conducted in the literature [46]. Interestingly, the annealing of Ti_3_C_2_T_x_-MXene above 773 K may induce ferromagnetism, as noted by another literature work [53]. It is envisaged to continue the present studies for higher temperatures (above 300 K) to observe the possible changes in magnetic properties and behavior of Ti_3_C_2_T_x_-MXene.

The magnetoresistance properties of Ti_3_C_2_T_x_-MXene show repeatability for use as sensors that can measure space environment magnetic fields. A direct use case for such sensors would be ambient magnetic field sensing for onboard electronics and systems or for surrounding environment monitoring on space missions where the temperatures may drop well below 273 K; e.g., on the surface of Europa (moon of Jupiter), the temperature can be as low as 93 K [54].

## 4. Conclusions

The significant outcomes of the present paper are the extensive experimental studies of the composition, structure, magnetization and thermal and magnetoresistive properties of Ti_3_C_2_T_x_-MXene and their temperature evolution. These outcomes include the first experimental reporting of the temperature dependence of the specific heat of Ti_3_C_2_T_x_-MXene, which reveals a metallic character of this material. The thermal analysis and mass spectroscopy data revealed the predominance of hydroxyl-like terminating groups, and the XRD study showed that the MXene material obtained is a single OH-layer terminated Ti_3_C_2_OH.

The temperature dependence of the specific heat of the Ti_3_C_2_T_x_-MXene studied indicates no phase transition in the measurement region of 2 K to 300 K and reveals a metallic character of the material with a Debye temperature about three times smaller than that for the parent MAX phase compound, Ti_3_AlC_2_. Also, the Sommerfeld coefficient of the electronic contribution to the specific heat is nearly two times smaller than that for the parent Ti_3_AlC_2_ material. The specific heat value of Ti_3_C_2_T_x_-MXene, close to room temperature, is more than an order of magnitude higher than that of the prominent 2D nanomaterial graphene.

The temperature variation of electrical resistivity shows a metallic-like behavior, i.e., a decrease with decreasing temperature, with an upturn at low temperatures, below 100 K, where a magnetoresistance of negative sign appears. The temperature cycling studies show a reduction in the electrical resistivity after the heating of the sample to 350 K and the subsequent cooling.

The straight-line magnetization curves obtained for various temperatures reveal the Pauli paramagnetism of the material, which is consistent with our specific heat results and the observations reported in the literature. The negative magnetoresistance of Ti_3_C_2_T_x_-MXene observed for low temperatures is attributed to spin-dependent phenomena. This could be expected as the calculations revealed a large slope of DOS at the Fermi level, which tentatively explains the negative sign of magnetoresistance as related to spin-dependent effects.

The presence of water molecules or hydroxyl groups during the synthesis and processing of Ti_3_C_2_T_x_-MXene affects its properties and their evolution with temperature, which was observed in the measurements. Water-free methods of synthesis reported in the literature might help to avoid such influence on the physical properties of Ti_3_C_2_T_x_-MXene in future work. Nevertheless, the physical properties measured in the presence of hydroxyl groups can still provide repeatable patterns for making observations (low-temperature electrical resistivity), possibly allowing Ti_3_C_2_T_x_-MXenes to be used for space applications in the near future.

## Data Availability

The datasets generated during and/or analyzed during the current study are available from the corresponding author on reasonable request.
